# The Effect of *Rubus idaeus* Polyphenols Extract in Induced Endometriosis in Rats

**DOI:** 10.3390/molecules29040778

**Published:** 2024-02-08

**Authors:** Elena-Mihaela Jianu, Raluca Maria Pop, Luciana Mădălina Gherman, Floricuța Ranga, Antonia-Mihaela Levai, Vasile Rus, Sorana D. Bolboacă, Roxana-Adelina Ștefan, Mădălin Mihai Onofrei, Ionel-Daniel Nati, Ioana Alexandra Stoia, Paul-Andrei Ștefan, Carina Mihu, Carmen Mihaela Mihu

**Affiliations:** 1 Histology, Department of Morphofunctional Sciences, “Iuliu Haţieganu” University of Medicine and Pharmacy, Victor Babeș, No 8, 400012 Cluj-Napoca, Romania; jianu.mihaela21@gmail.com (E.-M.J.); roxanalupean92@gmail.com (R.-A.Ș.); madalinonofrei99@gmail.com (M.M.O.); carmenmihu2004@yahoo.com (C.M.M.); 2 Pharmacology, Toxicology and Clinical Pharmacology, Department of Morphofunctional Sciences, “Iuliu Haţieganu” University of Medicine and Pharmacy, Victor Babeș, No 8, 400012 Cluj-Napoca, Romania; mihu.carina@elearn.umfcluj.ro; 3 Experimental Centre, “Iuliu Haţieganu” University of Medicine and Pharmacy, Louis Pasteur, No 6, 400349 Cluj-Napoca, Romania; 4 Food Science and Technology, Department of Food Science, University of Agricultural Science and Veterinary Medicine, Calea Mănăștur, No 3-5, 400372 Cluj-Napoca, Romania; floricutza_ro@yahoo.com; 5 Obstetrics and Gynecology, Department of Mother and Child, “Iuliu Hatieganu” University of Medicine and Pharmacy, Victor Babeș, No 8, 400012 Cluj-Napoca, Romania; levai.antonia@yahoo.com (A.-M.L.); nati.ionel@yahoo.com (I.-D.N.); 6 Department of Cell Biology, Histology and Embryology, University of Agricultural Sciences and Veterinary Medicine, Calea Mănăștur, No 3-5, 400372 Cluj-Napoca, Romania; vasile.rus@usamvcluj.ro; 7 Department of Medical Informatics and Biostatistics, “Iuliu Haţieganu” University of Medicine and Pharmacy, Louis Pasteur, No 6, 400349 Cluj-Napoca, Romania; sbolboaca@umfcluj.ro; 8 Obstetrics and Gynaecology Department County Emergency Hospital, Clinicilor, No 3-5, 400006 Cluj-Napoca, Romania; stoia.ioana04@yahoo.com; 9 Anatomy and Embryology, Department of Morphological Sciences, “Iuliu Haţieganu” University of Medicine and Pharmacy, Victor Babeș, No 8, 400012 Cluj-Napoca, Romania; stefan_paul@ymail.com

**Keywords:** endometriosis, dienogest, *Rubus idaeus*, matrix metalloproteinase-2, matrix metalloproteinase-9, transforming growth factor beta 1

## Abstract

Endometriosis is a common gynecological condition with a complex physio-pathological background. This study aimed to assess the role of *Rubus idaeus* leaf extract (RiDE) as a potential therapeutic agent in reducing the size of the endometriotic lesions and modulate the plasma expression of MMP-2, MMP-9, and TGF-β1. The endometriotic lesions were induced in a rat model by the autologous transplant of endometrium. Thirty-six female rats, Wistar breed, with induced endometriosis, were divided into four groups and underwent treatment for 28 days. The CTRL group received 0.5 mL/day of the vehicle; the DG group received 1 mg/kg b.w./day dienogest; the RiDG group received 0.25 mL/kg b.w./day RiDE and the D+RiDG group received 1 mg/kg b.w./day dienogest and 0.25 mL/kg b.w./day RiDE, respectively. Rats’ weight, endometriotic lesion diameter and grade, and plasma levels of MMP-2, MMP-9, and TGF-β1 were assessed before and after treatment. The administration of RiDE in association with dienogest vs. dienogest determined a lower weight gain and a reduction in diameter of the endometriotic lesions. RiDE administration restored MMP2 and MMP9 plasma levels to initial conditions. *Rubus idaeus* extract may help in reducing dienogest-associated weight gain, lower the size of endometriotic lesions, and have anti-inflammatory effects through MMP2 and MMP9 reduction.

## 1. Introduction

Endometriosis is an increasingly common pathology, considered by many researchers as the disease of the century [1]. World Health Organization (WHO) estimates that 10% of women of reproductive age worldwide are affected by this condition [2]. Endometriosis is a chronic estrogen-dependent disease defined by the histological presence of functional endometrial glands and stroma in ectopic areas, outside the uterine cavity [3]. Endometriosis lesions are characterized by a chronic inflammatory state, with increased infiltration of immune cells, which release various pro-inflammatory mediators, such as interleukin-1β, tumor necrosis factor-α, and prostaglandins [4].

A variety of factors have been suggested to contribute to the development of this condition, including retrograde menstruation, genetic predisposition, immunological dysregulation, and exposure to environmental stress factors [5]. Retrograde menstruation, which occurs when menstrual blood containing endometrial cells flows back into the peritoneal cavity, is widely accepted as a significant contributing factor in the development of endometriosis. Ectopic endometrial cells can attach and adhere to different tissues in the pelvis, resulting in the development of endometriosis lesions. The presence of inflammatory agents promotes the proliferation and invasion of endometrial cells, angiogenesis, and fibrosis, which are all facilitated by immune dysregulation [6].

Matrix metalloproteinase-2 (MMP-2) and matrix metalloproteinase-9 (MMP-9), often referred to as gelatinase B, are enzymes that are involved in the degradation of the extracellular matrix (ECM), which is a complex structure of proteins that envelops and sustains cells. MMP-2 and MMP-9 are involved in the invasion and dissemination of endometrial cells, which leads to the occurrence and development of endometriosis. Research has consistently demonstrated that women with endometriosis have higher levels of MMP-2 and MMP-9 expression in their blood, peritoneal fluid, and endometrium compared to healthy individuals [7].

Transforming growth factor-beta 1 (TGF-β1) is a complex cytokine that is essential for controlling cell proliferation, differentiation, and death. TGF-β1 in involved in several facets of the pathophysiology of endometriosis, with many processes resembling those of MMPs. TGF-β1 enhances the proliferation and viability of endometrial cells, hence facilitating the development and extension of endometriosis lesions. TGF-β1 amplifies the tendency of endometrial cells to infiltrate and attach to adjacent tissues, thus promoting the development of newly formed endometriotic lesions. Also, TGF-β1 further stimulates angiogenesis and facilitates endometriosis lesions’ development and their viability. TGF-β1 controls the synthesis and breakdown of extracellular matrix (ECM) elements, which play a role in tissue remodeling and the development of adhesions in endometriosis. TGF-β1 regulates the immune response in endometriosis by inhibiting the function of cytotoxic T cells and natural killer cells, which play an essential role in removing aberrant cells [8].

The first-line treatment option for endometriosis is hormonal therapy. It functions by inhibiting the synthesis of estrogen, restricting the proliferation of endometrial tissue, and relieving discomfort. Dienogest, a synthetic progestin widely used for the treatment of endometriosis, exhibits its therapeutic effects by inhibiting ovulation, and selectively blocking the activity of cyclooxygenase-2 (COX-2), thus inhibiting inflammation. Dienogest also can modulate endometrial growth and upregulate inhibin A protein synthesis, which suppresses the synthesis of estrogen, making it possible to prevent endometriosis’ ectopic development [9,10].

Aside from hormonal therapy and occasionally surgery, many adjunctive therapies might be beneficial for endometriosis, such as analgesics that can alleviate pain, dietary adjustments, or complementary and alternative medicine (CAM) including acupuncture, yoga, and herbal supplements. Nevertheless, the available data are insufficient to ascertain the effectiveness of these therapies. *Rubus idaeus*, often known as raspberry, is a plant that possesses a substantial historical background of customary utilization in several societies. The extract has attracted considerable scientific attention because of its wide range of pharmacological qualities, such as its ability to reduce inflammation and function as an antioxidant [11,12,13,14].

The different raspberry extracts that are obtained from the foliage, stems, seeds, or fruits of the plant are highly concentrated reservoirs of bioactive compounds [15,16,17]. Specifically, the chemical composition of *Rubus idaeus* leaf extracts includes polyphenols, specifically ellagitannins and flavonoids [18,19,20]. Ellagitannins, which include ellagic acid and sanguiin H-6, are potent antioxidants that shield cells from harm by free radicals [21]. Flavonoids, such as quercetin, kaempferol, and myricetin, possess antioxidant characteristics and have been associated with several health benefits, including anti-inflammatory and anti-cancer qualities [22]. Additionally, leaf raspberry extract has many bioactive parts, including phenolic acids like gallic acid and chlorogenic acid, which help make it an antioxidant and an anti-inflammatory [23]. Furthermore, it includes volatile chemicals such as linalool, geraniol, and caryophyllene, which can provide flavor and fragrance advantages while also demonstrating antibacterial characteristics [15].

*Rubus idaeus* leaf extract (RiDE) was also listed as having antidiabetic and antihyperlipidemic activity by improving the body’s response to insulin, decreasing the levels of glucose in the blood, and reducing lipid profiles. *Rubus idaeus* leaf extract inhibits enzymes such as lipases and downregulates the transformation of triglycerides into free fatty acids. Furthermore, studies have demonstrated that RiDE effectively promotes the absorption of cholesterol by cells, facilitating its elimination from the body [24,25].

The RiDE aids in protecting cells from oxidative stress and DNA damage, both of which contribute to the onset and proliferation of cancer. It effectively eliminates free radicals, inhibits the activity of enzymes that participate in DNA methylation, and offers further protection to cells by blocking the binding of carcinogens to DNA, hence preventing mutations. Raspberry leaf extract also inhibits the uncontrolled proliferation of cancer cells, thereby preventing their excessive division and unregulated expansion by possessing the capacity to interfere with the mechanisms that enable the invasion and spread of cancer cells [26,27]. Lung, laryngeal, breast, and colorectal cancers are among the illnesses that have benefited from the anti-inflammatory and cytotoxic activity of RiDE [28,29,30].

Our study aimed to evaluate the role of RiDE’s effects in reducing the size of the endometriotic lesion in an in vivo rat experimental model, and its influence upon MMP-2, MMP-9, and TGF-β1 plasma levels, known as possible modulators of inflammation processes.

## 2. Results

### 2.1. High-Performance Liquid Chromatography–Mass Spectrometry (HPLC-MS) Analysis of Polyphenols from Rubus idaeus Extract

**Figure 1:**
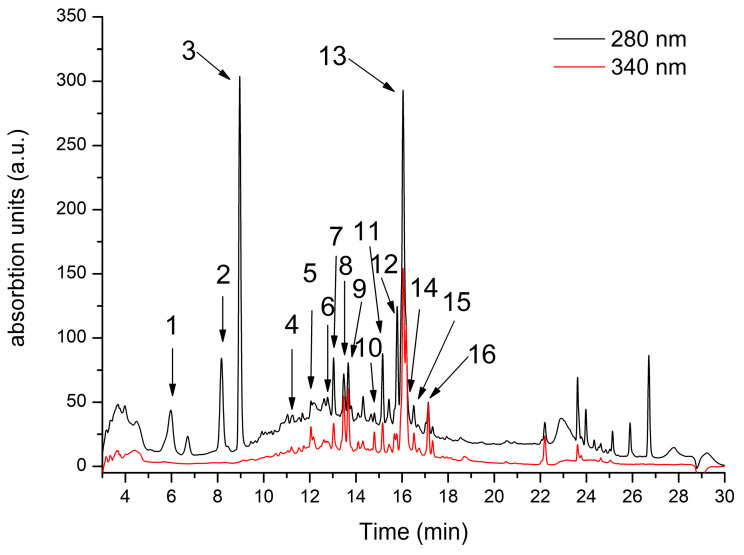
*Rubus idaeus* HPLC chromatogram registered at 280 nm (black line) and 340 nm (red line). For peak assignment, see Table 1.

Figure 1 shows the HPLC fingerprint of phenolic compounds found in RiDE water extract. In total, 16 compounds were identified, as described in Table 1. Hydroxybenzoic acid compounds were predominantly identified in RiDE, accounting for approximately 52% of total phenolics as quantified by LC-MS. Flavonols, flavanols, and hydroxycinnamic acids were also present at significant quantities, accounting for approximately 26%, 14%, and 8% respectively.

### 2.2. Preliminary Studies in Endometriosis Induction

**Figure 2:**
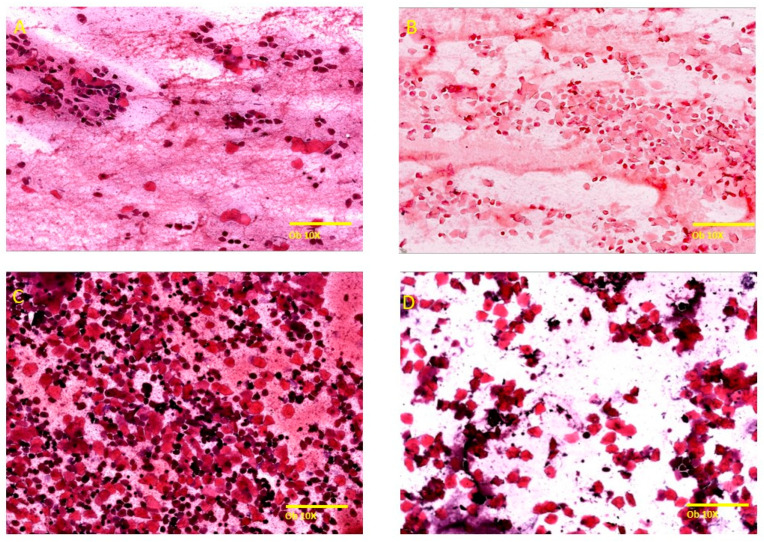
Representation of the stages of the menstrual cycle in rats under Hematoxylin–Eosin staining (Ob. 10×, scale bar 200 μm). (**A**) Proestrus: small, nucleated epithelial cells with uniform appearance, observed individually on the surface of the smear, low number of large epithelial cells and keratinized anucleated cells, the presence of a low number of neutrophils. (**B**) Estrus: predominantly anucleated keratinized epithelial cells, frequent bacteria adhered to the cells or free on the smear, cellular relicts, occasionally neutrophils in late estrus. (**C**) Metestrus: anucleated keratinized epithelial cells with frequent neutrophils, polymorphonuclear leukocytes, leukocyte infiltrate. (**D**) Diestrus: low cellularity, keratinized anucleated epithelial cells, frequent neutrophils with small, nucleated cells, blood material and mucus.

The vaginal secretion of female rats consists of three types of cells including nucleated epithelial cells, leucocytes, and anucleated cornified epithelial cells [31]. The four stages of the estrous cycle were identified based on their cytological specific criteria (Figure 2), specifically based on the proportions of these cells present in the vaginal secretion.

**Figure 3:**
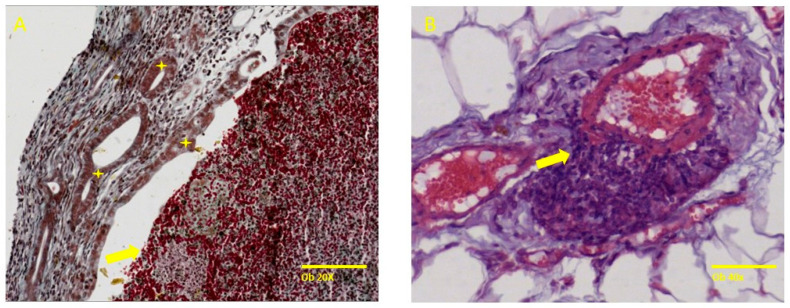
Specific histological findings such as endometrial cystic glands, some filled with a chocolate-like fluid and surrounding stroma with an increased number of capillaries. (**A**) represents specific endometrial glands (yellow star) and necrotic hemorrhagic content in the gland lumen (yellow arrow) and (**B**) shows endometrial stromal cells invading the tunica media of an artery (black arrow). Trichrome stain, ob 20× (scale bar 100 µm), Hematoxylin–Eosin ob 40× (scale bar 50 µm), respectively.

The induction of endometriosis, performed in accordance with the menstrual cycle phases, indicates that the prevalence rate was higher in the metestrus/diestrum stage (four out of seven) as compared with the proestrus/estrus stage (two out of seven). The endometriotic lesions showed typical histologic alterations of endometriotic lesions, such as the presence of specific endometrial glands, some cystic and filled with hemorrhage and necrosis, and endometrial stroma invading the structure of blood vessels or organs (Figure 3).

### 2.3. Macroscopic Evidence of Endometriotic Tissue

**Figure 4:**
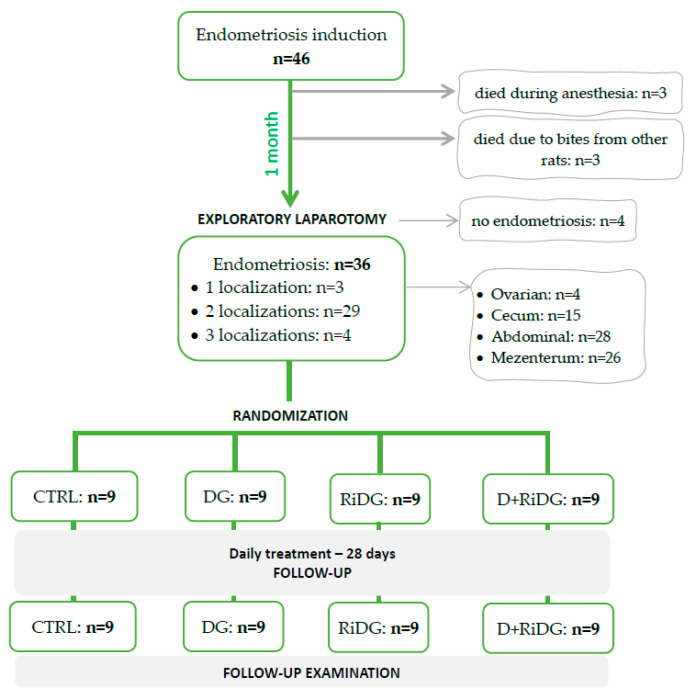
The flowchart from endometriosis induction to randomization and evaluation. CTRL = control group, DG = dienogest group, RiDG = *Rubus idaeus* leaf extract group, and D+RiDG = dienogest + *Rubus idaeus* leaf extract group.

Endometriosis was successfully induced in 40 out of 46 rats, followed by random allocation to the treatment and follow-up evaluation (Figure 4).

As expected, the weights of rats were increased in the follow-up, with the lowest increase in the CTRL group (Table 2).

The arithmetic mean diameter of the lesions is preserved in the CTRL group, while in treated groups, the values decreased with the most evident effects seen in D+RiDG (Table 2).

The grade of lesions remained in most cases unchanged (Table 2), with no statistically significant association between group and follow-up changes in lesion grade (χ^2^ = 3.2, *p*-value = 0.3563). We did not observe any increase in the lesion grade in the DG and D+RiDG groups (Table 2).

### 2.4. Effect of Rubus Idaues Leaf Extract on Plasma Levels of Matrix Metalloproteinases and Transforming Growth Factor Beta 1

**Figure 5:**
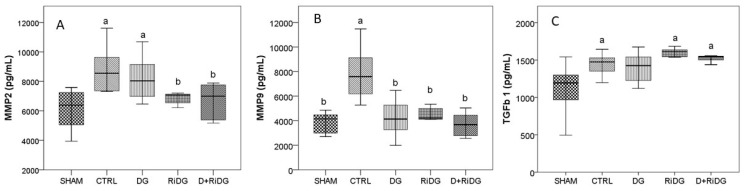
Plasma levels of matrix metalloproteinases ((**A**) MMP2 and (**B**) MMP9) and of transforming growth factor beta 1 ((**C**) TGF-β1) by groups. The boxplots show the variation of investigated parameters where the midline represents the median values while the extreme lines indicate the value of the first and third quartile. Significant values were considered at *p* < 0.05 versus the SHAM group, a and b had *p* < 0.05 versus the CTRL group.

Significantly higher levels of plasma MMP2 (*p* = 0.008), MMP9 (*p* = 0.001), and TGF-β1 (*p* = 0.053) were determined in the CTRL group when compared with the SHAM group (day 0) (Figure 5), supporting the successful induction of endometriosis in rats. The administration of dienogest succeeded in restoring the levels of MMP9, having statistically significant lower values (*p* = 0.007) than CTRL and no statistical difference when compared to the SHAM group. The administration of dienogest tended to reduce MMP2 and TGF-β1 levels as well, but with no statistical difference. The administration of RiDE significantly reduced MMP2 levels (*p* = 0.017) and MMP9 levels (*p* = 0.008) as compared to CTRL, restoring the levels similarly to the initial condition as observed when compared to the SHAM group. The administration of RiDE did not influence TGF-β1 level (Figure 5).

RiDE administration in addition to dienogest therapy succeeded in restoring the MMP2 and MMP9 values to the initial condition, with no statistically significant values as compared with SHAM, while in the case of TGF-β1, the plasma level showed no effect.

## 3. Discussion

The ectopic endometrial tissue responds to hormonal changes during the menstrual cycle, causing inflammation, dysmenorrhea, dyspareunia, persistent pelvic pain, irregular uterine bleeding, and infertility, thus endometriosis is considered a serious medical condition that has a major impact on the patient’s quality of life [32,33].

The mechanism of pain and inflammation in endometriosis is complex, not fully understood, and involves multiple factors such as chronic inflammation, neuroplastic changes, noxious stimuli, or peritoneal adhesions. Endometriosis can be treated clinically, surgically, or with both. The typical medicines, mainly hormonal therapy, cause disruptions to the menstrual cycle, and when the drugs are stopped, the pathology returns. The surgeries, when aiming to be curative or to remove adhesions, are often mutilating even though they can be straightforward. It is crucial to conduct experimental research using treatment approaches that can elucidate the pathophysiology of endometriosis. More insights into the degradation process of the extracellular matrix and further into the invasion and dissemination processes of endometrial cells will certainly help us to discover a more effective agent in the management of endometriosis. Thus, we aimed to identify alternative or complementary treatments to hormonal therapy to reduce its side effects. Consequently, RiDE’s effect on endometriosis lesions was approached both macroscopically and at the molecular level. The macroscopic examination targeted the evidence of endometriotic lesions such as weight, diameter, and grade, while the quantification of MMP2, MMP9, and TGF-β1 at the molecular level targeted the process of degradation of the extracellular matrix as well as the cell proliferation process.

Therefore, following the first aim, the first step of the current study was to mimic human endometriosis by using an autologous endometriosis rat model without ovariectomy or hormonal supplementation to obtain specific lesions. The preliminary study group was chosen on purpose to determine if the phase of the estrous cycle at the point of surgical induction may influence the development of endometriotic lesions. Because the proestrus and metestrus phases may last only up to a few hours [31], we deliberately chose to group the female rats in proestrus/estrus, and metestrus/diestrus, respectively. According to Cora and colleagues’ [34] criteria for staging the estrous cycle using vaginal smears, we have concluded that female rats in metestrus/diestrus, in our preliminary group, were more likely to develop endometriotic lesions. Disparate findings have been reported over the years regarding the most favorable estrous cycle phase appropriate for endometriosis induction to reach the highest probability of obtaining specific lesions. Kiani et al. [35] and Schreinemacher et al. [36] found no correlation between the timing of endometriosis induction in the estrous cycle phase and the latter endometriotic lesion achievement. On the other hand, other studies [37,38] are in favor of proestrus and estrus phases to perform the implants, reporting a higher chance of obtaining endometriotic lesions. A higher prevalence rate along with the volume of endometriotic lesions was also reported by Uchida and colleagues [39]. Within our study, the endometriosis induction success rate was 45.62%, which is consistent with the one reported by Kiani et al. [35] of 41.5%.

Variations in body weight were associated with the presence of endometriosis or hormonal-specific treatment. Weight loss due to chronic pelvic pain [40] or weight gain secondary to dienogest [41] have been reported. In our study, the female rats in the control group showed the lowest weight gain one month after endometriosis induction and in evolution when receiving no active medication (*p* < 0.0003). This phenomenon may be explained by the fact that chronic pain and inflammation induced by the presence and evolution of endometriotic tissue in the absence of treatment may determine the loss of appetite and decreased weight gain. At the end of the treatment, female rats in DG showed the highest weight increment with a median of 234 g (231 to 239), compared to RiDG 218 g (217 to 221) and D+RiDG 224 g (219 to 228) (*p* < 0.0001). Weight gain according to the percentile under treatment was expected, but the association of RiDE with dienogest in D+RiDG determined a lower weight gain in comparison to the female rats in DG, thus implying that RiDE may help in reducing dienogest-associated weight gain.

The lesions’ mean diameter in the CTRL group was preserved or tended to expand because, without specific treatment, endometriosis is a chronic evolving illness showing continuous growing and spreading. In female rats from all the treated groups, the values of the arithmetic mean diameter of the lesions decreased, with the most evident effects in the D+RiDG (Table 2), but with no statistical significance between groups, indicating that substances administration should be prolonged.

The second aim of this study was to identify alternative or complementary treatments to hormonal therapy to reduce its side effects. The approach was based on reducing inflammation, knowing its major role in endometriosis progression [42]. The uncontrolled cascade of inflammatory processes can lead to the upregulation of metalloproteinases, TGF-β, prostaglandins, chemokines, and cytokines [43]. Among these, the relationship between different diseases and the levels of MMPs is very important, since their activities are seen as elevated plasma, serum, or tissue levels in illnesses like cancers, diabetes, cardiovascular diseases, and neurodegenerative diseases [44,45,46,47,48,49,50]. The heightened activity of MMP-2 and MMP-9 plays a significant role in several important aspects of endometriosis as well. Specifically, the mechanism of action of MMP-2 and MMP-9 involves the degradation of the extracellular matrix (ECM), which facilitates the infiltration and attachment of endometrial cells to neighboring tissues, ultimately resulting in the development of endometriosis lesions [51]. Also, angiogenesis is facilitated by MMP-2 and MMP-9, which stimulate the development of newly formed blood vessels. These arteries provide oxygen and nutrition to endometriosis lesions, hence supporting their growth and survival [52,53]. Tissue remodeling is promoted by MMP-2 and MMP-9, which play a role in the generation of adhesions, the fibrous tissue that can cause tissues to adhere together in endometriosis [54]. Also, other factors can influence the levels of MMP-2, MMP-9, and TGF-β1 in endometriosis, including estrogen and progesterone, which are the primary sex hormones. Estrogen typically enhances the expression of MMP-2, MMP-9, and TGF-β1, while progesterone tends to decrease it. Inflammatory mediators, such as cytokines and growth factors, can also stimulate the production of MMP-2, MMP-9, and TGF-β1 in endometriosis. Additionally, certain genes that regulate MMP-2, MMP-9, and TGF-β1 may contribute to the elevated expression of these enzymes in endometriosis.

To date, there are not many studies focused on the utilization of anti-MMP compounds in patients with endometriosis. Most of the studies discuss the use of MMPs as biomarkers underlying drugs’ ability to reduce their expression [7]. Our study demonstrated that the administration of RiDE may be an important agent for treating endometriosis, especially by reducing the MMPs levels. Specifically, it was observed that the administration of RiDE in rats with endometriosis was able to restore MMP2 and MMP9 values to normal conditions. The mechanism of action by which RiDE reduces MMPs levels is yet to be elucidated. When various molecules and cytokines such as MMPs, VEGFs, hypoxia-inducible factor-1α (HIF-1α), IL-2, IL-27 are dysfunctional, they enhance both angiogenesis and the development of endometriotic lesions [55,56]. Also, the growth and progression of endometrial stromal cells are influenced by the alteration of different pathways, such as NF-κB, Wnt/β-catenin, JAK/STAT, COX2/PGE2, and TGF-β upregulation. These abnormalities can lead to the expression of tumor-like traits in endometrial stromal cells, such as the overproduction of ROS, inflammatory reactions, immune system activation, angiogenesis, proliferation, fibrosis, apoptosis, and autophagy [7,55,57,58,59]. Further, Tcf/β-catenin targets both MMP-9 and MMP-2 genes. Consequently, it was observed that in endometrial epithelial cells from patients with endometriosis, Cyclin D1, a Tcf/β-catenin target gene, had significantly higher mRNA expression in the mid-secretory phase [60]. In addition to angiogenesis, invasion, tissue organization, and cell survival, MMPs may also be involved in the regulation of cytokines, other MMP family members, and latent growth factors [61].

However, studies in the literature have already suggested that the daily optimal consumption of fruits and vegetables high in polyphenol compounds decreases the risk of developing specific chronic and degenerative diseases with an inflammatory component, via MMPs regulation [62,63,64]. Accordingly, multiple studies have examined the chemical composition of the raspberry leaf extract, uncovering a wide range of bioactive compounds that have prompted research into its potential uses in several diseases, especially those related to inflammation and metabolic disorders [22,65]. More specifically, the antioxidant and anti-inflammatory effects of the phenolic compounds extracted from *Rubus idaeus* leaves can be attributed to the rich composition of the extract in ellagic acid, quercetin, and kaempferol derivatives, as well as catechins. The effects of ellagic acid have been described to regulate multiple pathways such as proinflammatory agents’ inhibition [66], antioxidant response activation [67], fibrotic formation, growth factor, and adhesion molecules regulation [68,69]. Quercetin enacts an anti-inflammatory activity through the inhibition of the eicosanoid’s pathway via enzymatic and non-enzymatic arachidonic acid oxidation, which in turn can reduce the production of inflammatory mediators such as prostaglandins and leukotrienes [70,71]. Additionally, the anti-inflammatory effect of kaempferol is strongly related to its ability to suppress the release of IL-1β, IL-6, IL-18, and TNF-α [72], to increase the mRNA and protein expression of Nrf2-regulated genes [73], to inhibit the toll-like receptor 4 (TLR4) [74], and to inhibit COX1 and COX2 production [75,76]. The role of catechins in reducing inflammation is also well documented. Thus, catechins possess anti-inflammatory activities by regulating the activation/deactivation of different inflammatory and oxidative stress cell signaling pathways like nuclear factor-kappa B (NF-κB), transcription factor nuclear factor (erythroid-derived 2)-like 2 (Nrf2), mitogen-activated protein kinases (MAPKs) or signal transducer, and the activator of transcription 1/3 (STAT1/3) pathways [77]. Also, according to the literature, another possible effect of phenolics has been reported on epi transcriptomic pathways, such as the fact that m6A modification might modulate TGFβ signaling [78,79] as a result of its activation during the epithelial to mesenchymal transition process characteristic of endometriosis [80,81].

Nevertheless, the anti-inflammatory effect of RiDE is obtained due to the synergistic effects of various polyphenol compounds. That is, its beneficial effect is supported by its well-known utilization in traditional herbal medicine for centuries, having been used to treat many ailments associated with the female reproductive system, particularly during pregnancy, and to aid in childbirth, with long-lasting effects seen into the modern day [82,83].

## 4. Materials and Methods

### 4.1. Chemicals

Acetonitrile, acetic acid, and carboxymethylcellulose sodium (CMC) were purchased from Sigma Aldrich (St. Louis, MO, USA). Dienogest was purchased from Bayer Weimar GmbH und Co. KG (Weimar, Germany). The injectable anesthetics, ketamine and xylazine, were purchased from Biotur Exim SRL, Alexandria, Romania. The RiDE used in this study is a commercial product produced by Herbiolys Laboratoire Bio (Lardier-et-Valença, France), and it contains bio raspberry 3% leaf bud extract, vegetable glycerin and low mineralized spring water.

### 4.2. High-Performance Liquid Chromatography–Mass Spectrometry (HPLC-MS) Analysis of Polyphenols from Rubus idaeus Extract

The phenolic compounds’ separation was performed according to the protocol described by Pop et al. (2018) [24]. In short, an Agilent 1200 HPLC apparatus with a DAD detector connected to a 6110 single quadrupole MS was used for compound identification and quantification. The compound separation was achieved using a reverse-phase Eclipse XDB C 18 column (4.6 mm × 5.6 mm, 5 μm, Agilent Technologies, Santa Clara, CA, USA) using a gradient elution of (A) water + 0.1% acetic acid and (B) acetonitrile + 0.1% acetic acid for 30 min. The temperature was set at 25 °C at a flow of 0.5 mL/min. The gradient used was as follows (expressed as % B): 0 min, 5% B; 0–2 min, 5% B; 2–18 min, 5–40% B; 18–20 min, 40–90% B; 20–24 min, 90% B; 24–25 min, 90–5% B; 25–30 min, 5% B. The compound spectra were registered at 280 for phenolic acids and 340 nm for flavonols, respectively. Quantification was performed using calibration curves of different standard solutions of ellagic acid (R^2^ = 0.9978; LOD = 0.35 μg/mL, LOQ = 1.05 μg/mL), catechin (R^2^ = 0.9985; LOD = 0.18 μg/mL, LOQ = 0.72 μg/mL), chlorogenic acid (R^2^ = 0.9937; LOD = 0.41 μg/mL, LOQ = 1.64 μg/mL), gallic acid (R^2^ = 0.9978; LOD = 0.36 μg/mL, LOQ = 1.44 μg/mL) and rutin (R2 = 0.9981; LOD = 0.21 μg/mL, LOQ = 0.84 μg/mL). Hydroxybenzoic acids (peaks 1,2 3 and 9) were quantified as gallic acid equivalents; ellagic acid and its derivatives (peaks 7, 10, and 13) were calculated as ellagic acid equivalents; hydroxycinnamic acids (peaks 5 and 8) were quantified as chlorogenic acid equivalents, flavanols (peaks 4 and 6) were calculated a catechin equivalents, while flavonols (peaks 10, 12, 13, 15 and 16) were calculated as rutin equivalents. Compound identification was performed based on retention time, UV-Vis absorption spectra, and mass spectra by comparison with the ones obtained from standards, the Phenol-Explorer database, and data in the literature.

### 4.3. Animals

A total number of 60 young virgin female rats, Wistar breed, aged approximately 6–8 weeks, 145–160 g, were used during this experimental study. Before enrolment in the experiment, the female rats were subjected to the acclimatization process, over 10 days, the animals being kept (5 rats/cage) in a light/dark cycle of 12 h, having ad libitum access to water and food. The experimental procedure was accepted and approved by the Ethics Committee and the Veterinary Health Department (No 314/20.05.2022) and carried out following legislation on the use of laboratory animals, as well as with the animal care regulations in force.

### 4.4. Endometriosis Surgical Induction in Rats

#### 4.4.1. Preliminary Studies in Endometriosis Surgical Induction

Initially, 14 female rats were selected to be included in a preliminary study that aimed to establish which of the phases of the estrous cycle is the most appropriate to successfully induce endometriosis. Each female was subjected to a clinical examination to assess the vaginal opening and subsequently, daily vaginal smears were performed, to identify the four stages of the estrous cycle (proestrus, estrus, metestrus, and diestrus). Following this, the preliminary study group was subdivided into two subgroups: the proestrus/estrus subgroup (7 female rats) and metestrus/diestrus (7 female rats), respectively. All 14 female rats in the preliminary study underwent the experimental induction of endometriotic lesions. For the surgical induction of endometriosis, the animals were subjected to general intraperitoneal anaesthesia (ketamine 0.01 mg/kg and xylazine 0.01 mg/kg). Throughout the intervention, the animals were kept in aseptic conditions, and the body temperature was maintained at 37 °C by placing them on a heated plate.

**Figure 6:**
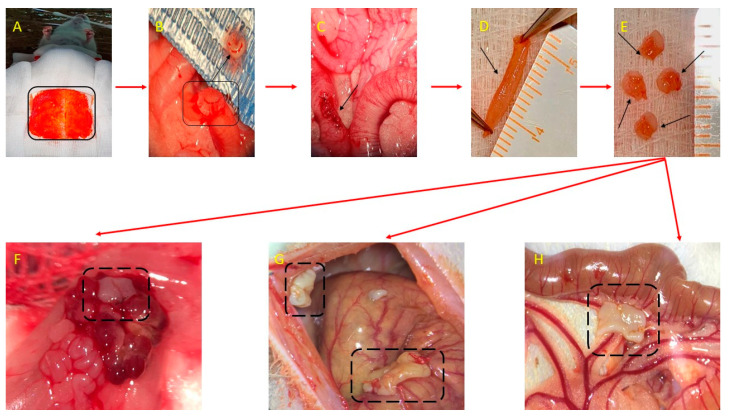
Surgical induction of endometriosis-autologous transplant: (**A**) Anesthesia, restraint of the animal, identification, and highlighting of the tissue sampling area. (**B**) A portion of tissue from the right uterine horn of the rat was removed, through a longitudinal opening. (**C**) Suture of the uterine horn after harvesting the endometrial tissue. (**D**) The portion of endometrial tissue harvested after longitudinal sectioning. (**E**) The 4 uterine fragments with a size of 2.5/3.5 mm, which are sutured at the level of the left ovary (**F**), the abdominal wall (the right side) (**G**), the cecum (**G**) and the mesentery (**H**).

The endometriosis induction surgery steps (Figure 6) were based on the initial protocol described by Vernon and Wilson for rats [84] and Cummings and Metcalf [85]. Accordingly, the animals were prepared for surgery by trimming the abdominal area, and then a median incision was made at the level of skin tissue and the abdominal muscle to expose the organs of the pelvic and abdominal cavities. The uterine horns were identified, and a piece of approximately 1.5 cm of the middle portion of the uterine right horn wall was harvested. The tissue block was split longitudinally and processed so that only the endometrium was retrieved and prepared in 4 implants with an approximate size of 2.5 *×* 3.5 mm from the excised uterus. In each rat, the implants were sutured at the level of the abdominal parietal peritoneum, to the surface of the left ovary, to the surface of the cecum, and the mesentery near the artery irrigating the cecum. The implants were sutured with the endometrium towards the peritoneal cavity using a using 8-0 Nylon monofilament suture thread. After applying the implants, the muscle layer on the abdomen was sutured using 6-0 Nylon monofilament suture thread and the skin tissue was sutured using 3-0 Nylon monofilament suture thread.

After the intervention, the rats were carefully monitored for the following days for signs of wound infection or acute wound complications such as dehiscence, evisceration, hernia, seroma, or hematoma.

One month later, during exploratory laparotomy, the macroscopic evaluation of the abdominal and peritoneal cavities indicated that female rats in the metestrus/diestrus phase at the time of the induction were more likely to develop specific endometriotic lesions. The endometriotic lesions were harvested, and trichrome and H&E stains were performed on the tissue specimen, and endometriosis was microscopically proven.

#### 4.4.2. Endometriosis Histopathological Assessment

Macroscopically dark red to brown fluid-cystic soft lesions were retrieved from the female rats in the preliminary group and were fixed in 10% formalin solution. After embedding in paraffin, 5 µm-thick tissue samples were prepared to be stained with Hematoxylin and Eosin and trichrome stain, and examined under a light microscope (Leica DM750 LED Phase Microscope ICC50W Camera Module). The presence of endometrial glands and endometrial stroma was assessed to confirm the diagnosis.

#### 4.4.3. Endometriosis Surgical Induction-Experimental Study

According to the results of the initial preliminary experiment, endometriosis was induced in another 46 female rats. Before surgery, each female rat was clinically examined to assess its health state. Next, surgical induction was performed during the metestrus/diestrus phase of the estrous cycle. During the entire surgical intervention, the animals were carefully monitored. During the follow-up, the rats were monitored daily for proper food and water intake and their behavior. Their weight was also recorded before endometriosis induction, at 4 weeks since endometriosis induction, and at 8 weeks (at the end of the experiment).

#### 4.4.4. Endometriosis Macroscopic Evaluation

**Figure 7:**
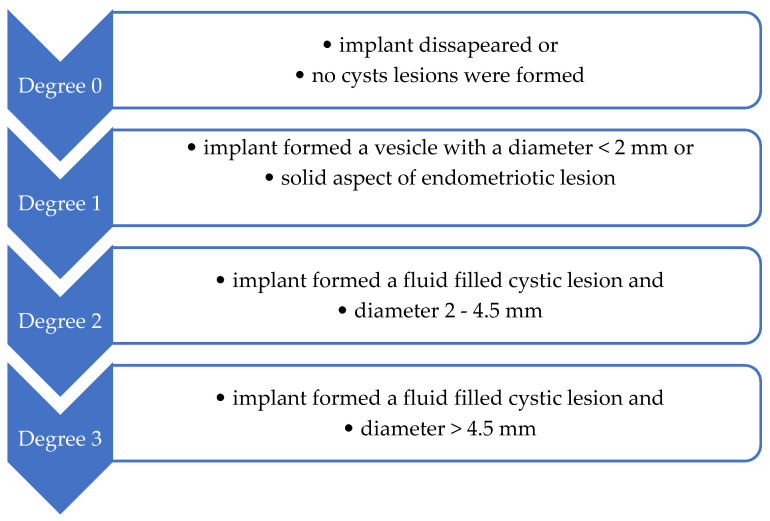
Endometriotic lesions’ classification, considering the degree of macroscopic growth (Quereda et al. [86]).

One month after the endometriosis surgical induction, the remaining 40 female rats were subjected to an exploratory laparotomy under general anesthesia as in the initial stage of the experiment. Macroscopic evaluation during the exploratory laparotomy aimed to evaluate the abdominal and pelvic cavities and assess the endometriosis-specific lesions. At this stage, the endometriosis was evaluated based on the correlation between macroscopic and microscopic aspects of the endometriotic lesions as induced in the preliminary study, the diagnosis of endometriosis being macroscopically assumed. For each female rat that developed macroscopic endometriotic lesions, the lesions were measured with a ruler and classified as exemplified in Figure 7. The mean of the degree of macroscopic growth was calculated for each female rat.

#### 4.4.5. Treatment Administration in Endometriotic Rats

The day after the exploratory laparotomy, the female rats with endometriosis were randomly divided into 4 groups, 9 rats/group as follows: control group (CTRL), dienogest group (DG), *Rubus idaeus* group (RiDG), and dienogest + *Rubus idaeus* group (D+RiDG). The CTRL group received 0.5 mL/day CMC, the DG group received dienogest (1 mg/kg b.w./day, where b.w. = body weight), the RiDG group received RiDE (0.25 mL/kg b.w./day), and the D+RiDG group received dienogest (1 mg/kg b.w./day) and RiDE (0.25 mL/kg b.w./day), respectively. All substances were administered orally by gavage. Dienogest was suspended in CMC before administration. The treatment was administered daily for 28 days.

At the end of the treatment period, the rats were euthanized with an overdose of anesthetics, and then the abdominal and pelvic cavities were evaluated to assess the evolution of the endometriotic implants under treatment.

### 4.5. Blood Samples and ELISA Analysis

In total, 2 mL of blood was drawn from the retroorbital sinus in the two steps of the experiment: before applying the implants and at the end of the treatment. The blood was collected using EDTA vacutainers and centrifuged at 2500 rpm for 10 min. Plasma was separated and then stored at −80 °C until analysis.

The plasma levels of matrix metalloproteinase 2 (MM2), matrix metalloproteinase 9 (MMP9), and transforming growth factor-beta 1 (TGF-β1) were measured using the enzyme-linked immunosorbent assay (ELISA) technique (Stat Fax 303 Plus Microstrip Reader, Minneapolis, MN, USA). The analysis was performed according to the instruction manuals provided by the commercially available kits (MMP2 and MMP9- Boster Biological Technology, USA and TGF-β1- FineTest-Wuhan Fine Biotech Co., Ltd., Wuhan, China). The results obtained from the rats before applying the implants are referred to in the text as the SHAM group.

### 4.6. Statistical Analysis

The diameter of lesion per rat was considered as the arithmetic mean of all lesions regardless of the location. The measurements were reported as median and (Q1 to Q2) (where Q1 is the 25th percentile and Q3 is the 75th percentile) since the variability of measurements was high within the groups. An overall comparison between groups was made using the Kruskal–Wallis test, followed by post-hoc analysis whenever statistically significant differences were observed. The differences within a specific group were tested with the Friedman test when more than two measurements were available and with the Wilcoxon test when two paired measurements were available. Numbers and percentages have been reported as summary statistics of qualitative variables. No statistical tests were applied to qualitative variables because the assumptions of the Chi-squared family tests were not accomplished. Data were analyzed with the Statistica program (v. 13, StatSoft, St Tulsa, OK, USA). For ELISA analysis, data were presented as mean ± standard deviation (SD). Further, an independent sample *t*-test was applied to check the statistical significance between different groups. *p*-values lower than 0.05 (*p* < 0.05) were taken into consideration.

## 5. Conclusions

We may conclude that the administration of RiDE has an anti-inflammatory effect, as observed by the reduction in endometriotic lesions and the reduction in both plasma MMP2 and MMP9 levels. The fact that the macroscopic examination yielded no statistically significant results indicates that the administration of RiDE over a longer period may lead to more significant clinical effects. The main strength of this study was the administration of RiDE in addition to dienogest treatment, miming the situations encountered in day-to-day life when most people take natural supplements without any medical advice. This information is valuable, offering important insights into the effects of plant administration concomitantly with drugs, since they can either have enhancing or reducing drug effects and can induce adverse reactions. This study also had some limitations. One limitation of this study was the short-term administration of dienogest and RiDE. Thus, the effects of the used substances should be monitored over a longer period. Another limitation was that tissue levels of MMPs and TGFβ1 were not determined. Thus, future studies will be needed to investigate whether the association of different concentrations of RiDE and lower doses of dienogest might be efficient in reducing the diameter of endometriotic lesions. Consequently, it could be explored whether dienogest’s dose-dependent side effects on long-term administration will be diminished by RiDE association. Secondly, the correlation between plasma and tissular levels of MMPs and TGFβ1 should be approached to offer a better perspective on the effects of RiDE in endometriosis etiopathogenesis and its adjuvant role in endometriotic lesions management.

## Figures and Tables

**Table 1:** LC–MS data and phenolic compounds’ identification in *Rubus idaeus* leaf extract.

Peak No.	R_t_ (min)	UV λ_max_ (nm)	[M+H]^+^ (m/z)	Phenolic Compound	Subclass	Concentration (μg/mL)
1	5.98	270	328, 171	Gallic acid-glucoside	Hydroxybenzoic acid	33.14 ± 0.67
2	8.17	280	155	2,4-Dihydroxybenzoic acid	Hydroxybenzoic acid	23.56 ± 2.02
3	8.96	280	301	4-Hydroxybenzoic acid-glucoside	Hydroxybenzoic acid	59.43 ± 7.19
4	11.25	280	579	Procyanidin dimmer I	Flavanol	24.18 ± 1.96
5	12.05	331	355	Chlorogenic acid	Hydroxycinnamic acid	10.34 ± 0.11
6	12.77	280	291	Catechin	Flavanol	27.33 ± 1.65
7	13.03	360, 280	435	Ellagic acid-arabinoside	Hydroxybenzoic acid	26.18 ± 1.46
8	13.47	330	343	Caffeic acid-glucoside	Hydroxycinnamic acid	21.55 ± 0.42
9	13.67	270	169	Vanilic acid	Hydroxybenzoic acid	13.89 ± 1.10
10	14.81	350, 255	611, 287	Kaempferol-diglucoside	Flavonol	8.68 ± 0.25
11	15.15	360, 280	465	Ellagic acid-glucoside	Hydroxybenzoic acid	2.08 ± 0.10
12	15.79	360, 260	611, 303	Quercetin-rutinoside (Rutin)	Flavonol	23.45 ± 1.67
13	16.04	360, 260	465, 303	Quercetin-glucoside	Flavonol	45.17 ± 2.03
14	16.18	360, 280	303	Ellagic acid	Hydroxybenzoic acid	40.69 ± 5.05
15	16.51	360, 260	478, 303	Quercetin-glucuronide	Flavonol	8.56 ± 0.11
16	17.14	350, 255	463, 287	Kaempferol-glucuronide	Flavonol	12.98 ± 0.69
				Total phenolics		381.29 ± 26.42

*Rubus idaeus* concentration (μg/mL).

**Table 2:** Weight, diameter, and grade of lesions, by groups, from baseline to end of treatment.

	CTRL	DG	RiDG	D+RiDG	Stat. (*p*-Value) *
Weight, g ^A^					
baseline	152 [149 to 153]	152 [147 to 159]	152 [149 to 154]	155 [148 to 158]	0.1 (0.9914)
1 month	174 [172 to 176]	179 [176 to 180]	175 [169 to 182]	178 [176 to 179]	3.8 (0.2822)
Follow-up	206 [204 to 211]	234 [231 to 239]	218 [217 to 221]	224 [219 to 228]	28.2 (<0.0001) ^a^
Stat. (*p*-value) **	16.0 (0.0003)	18.0 (0.0001)	18.0 (0.0001)	18.0 (0.0001)	
Diameter of lesion, mm ^A^					
preT	3.9 [3.5 to 7.75]	12.2 [7.7 to 13]	5.7 [5.2 to 9.2]	13.7 [13 to 15.2]	17.4 (0.0006) ^b^
postT	4 [3.4 to 7.65]	5.7 [3.5 to 6.35]	7.4 [3.6 to 8.4]	5.9 [4.5 to 6.9]	1.5 (0.6745)
Stat. (*p*-value) #	9 (0.0630)	2.7 (0.0077)	0.9 (0.3743)	2.7 (0.0077)	
PreT Grade ^B^					n.a.
0	18 (50.0)	16 (44.4)	18 (50)	19 (52.8)	
1	7 (19.4)	0 (0.0)	2 (5.6)	0 (0.0)	
2	11 (30.6)	15 (41.7)	14 (38.9)	10 (27.8)	
3	0 (0.0)	5 (13.9)	2 (5.6)	7 (19.4)	
PostT Grade ^B^					n.a
0	18 (50)	16 (44.4)	18 (50)	19 (52.8)	
1	5 (13.9)	5 (13.9)	7 (19.4)	4 (11.1)	
2	13 (36.1)	12 (33.3)	11 (30.6)	12 (33.3)	
3	0 (0.0)	3 (8.3)	0 (0)	1 (2.8)	
Grade change ^B^					n.a.
Grade 0	17 (47.2)	16 (44.4)	17 (47.2)	19 (52.8)	
Unchanged (Grade 1–3)	15 (41.7)	13 (36.1)	11 (30.6)	7 (19.4)	
Decreased	1 (2.8)	7 (19.4)	7 (19.4)	10 (27.8)	
Increased	3 (8.3)	0 (0.0)	1 (2.8)	0 (0.0)	

^A^ Data are reported as median (Q1 to Q3), where Q1 is the 25th percentile and Q3 is the 75th percentile. ^B^ Data are reported as no. (%). * Kruskal–Wallis test; ** Fridman ANOVA test; # Wilcoxon test; preT = before treatment; postT = post treatment. ^a^ CTRL vs. DG: *p*-value < 0.0001. CTRL vs. D+RiDG: *p*-value = 0.0108. DG vs. RiDG: *p*-value = 0.0093. ^b^ CTRL vs. D+RiDG: *p*-value = 0.0009. D-RiDG vs. RiDG: *p*-value = 0.0203; n.a. = not applicable.

## Data Availability

The datasets used and/or analysed during the current study are available from the corresponding author on reasonable request.
